# Assessing trends in clonorchiasis incidence via prescription data analysis in South Korea

**DOI:** 10.1186/s12889-025-23893-9

**Published:** 2025-08-21

**Authors:** Taeksang Lee, Jun Hyun Lee, Hanna Jin, Yun Kyung Lee, Hyun Beom Song

**Affiliations:** 1 Department of Tropical Medicine and Parasitology, Institute of Endemic Diseases, Seoul National University College of Medicine, Seoul, Republic of Korea; 2 Department of Biomedical Sciences, Seoul National University College of Medicine, Seoul, Republic of Korea; 3 Catholic Kwandong University Medical School, Gangneung-si, Republic of Korea

**Keywords:** Clonorchis sinensis, Republic of Korea, Parasitic diseases, Drug prescription record, Infectious disease epidemiology

## Abstract

**Background:**

While most parasitic diseases are being eradicated, clonorchiasis remains endemic and continues to pose a significant public health burden in South Korea. The prevalence has been monitored through nationwide or endemic area surveys and sentinel surveillance systems, but surveys based on stool examinations are resource-intensive and unable to determine incidence rates and trends unless conducted nationwide. Indeed, the nation-wide surveys have not been conducted since 2012 and endemic area surveys and sentinel surveillance systems also have limitations.

**Methods:**

We estimated the number of annual nationwide clonorchiasis cases by utilizing the National Health Insurance Service drug prescription information dataset for 2002–2023 and disease-specific prescription pattern. We then assessed trends in the overall incidence rates as well as sex-, age-, and region-specific incidence rates of clonorchiasis.

**Results:**

Our analysis estimated that the incidence rate of clonorchiasis in Korea showed a continuous decreasing trend over 20 years. Sex-, age-, and region-specific analysis showed that males, individuals aged 40 or older, and those residing in Gyeongsang and Jeolla provinces tend to have higher incidences of clonorchiasis. This trend aligns with findings from existing surveys and other available statistics.

**Conclusions:**

Our study highlights this novel method to estimate clonorchiasis incidence trend by utilizing publicly available drug information data. This approach will provide valuable insights for a fast and cost-effective way to estimate incidence rates and investigate trends for diseases with specific treatment options.

**Supplementary Information:**

The online version contains supplementary material available at 10.1186/s12889-025-23893-9.

## Introduction

Clonorchiasis prevalent endemic parasitic infections in the Republic of Korea [1–3]. It is caused by the liver fluke *Clonorchis sinensis* and is transmitted to humans through the consumption of raw or undercooked freshwater fish infected with the parasite’s metacercariae [4]. Due to its association with dietary habits, it is known to be disproportionately prevalent among older men living in river basin areas [5, 6]. The fluke resides in the biliary ducts for decades, causing chronic inflammation, tissue damage, and even cancer, which has been classified as a group I biological carcinogen by the International Agency for Research on Cancer. Several studies have reported an association between clonorchiasis and the incidence of extrahepatic cholangiocarcinoma [7]. Notably, South Korea is known to have the highest incidence rate of cholangiocarcioma, especially extrahepatic cholangiocarcinoma [8–10]. The persistent prevalence of clonorchiasis in the Korean population poses a significant public health burden.

The prevalence of clonorchiasis has been monitored through various surveys and surveillance system. The Korean government initiated a nation-wide intestinal helminth survey in 1971 to collect samples from more than 2% of the national population and conduct stool examinations to systematically monitor major helminthiasis. So far, a total of eight surveys have been conducted, with the most recent one in 2012 [11–13]. Following this, the Korean Disease Control Agency (KDCA) started monitoring major river basin areas as endemic regions for helminthiasis every year [14–17]. Stool examinations were conducted in regions where local public health centers voluntarily participated. In addition, the Korea Association of Health Promotion (KAHP) provides helminthiasis statistics with data based on their health checkup service [18]. They provide stool examination as an option for health checkup programs. Moreover, the Korean government has been continuously monitoring the number of clonorchiasis cases through a sentinel surveillance system since 2011. This approach is based on the Infectious Disease Control and Prevention Act, which was completely amended in 2009. Under this act, clonorchiasis and several other helminthiasis were designated as targets for sentinel surveillance [19]. These data provide valuable insight into clonorchiasis management in Korea, yet there remains a lack of methods to estimate incidence rates in nationwide population.

Alternatively, the use of national health insurance data can complement these existing methods. South Korea has a universal health insurance system, with 97% of the population covered by the National Health Insurance Service (NHIS), mandated by the government, and the remaining 3% covered by medical aid [20]. The NHIS, which manages reimbursements to medical providers, annually releases three separate sets of anonymized data for randomly selected 1 million individuals (about 2% of the entire population) as part of the National Focus Open Data, consisting of medical treatment history, drug prescriptions and health checkups. Although diagnostic codes in the medical treatment history dataset can be utilized to investigate disease trends [21], they overestimate the incidence, as these diagnostic codes are also assigned to the claims where diagnostic tests were performed for suspicious individuals. To overcome these limitations, we utilized disease-specific prescription data to estimate the incidence of clonorchiasis.

Praziquantel is a drug used to treat trematode and cestode infections and cannot be obtained without a doctor’s prescription in South Korea. Since it is prescribed under a distinct dosing regimen for clonorchiasis and prescription patterns are recorded in the NHIS database, praziquantel prescription records meeting specific criteria can serve as a proxy for disease incidence. This study leverages this relationship to develop and validate a method for estimating the incidence rates of clonorchiasis by using the prescription pattern. We demonstrate this by analyzing dataset from 2002 to 2023 to acquire estimated clonorchiasis case number, calculating annual incidence rates for each sex, age group and region, and comparing our estimates with existing surveillance data to validate the method’s reliability. We aim to establish prescription pattern analysis as a complementary tool for disease surveillance, particularly valuable for conditions where traditional surveillance methods prove impractical or cost prohibitive. The methodology presented here may be adaptable to other diseases with specific treatment patterns, offering a scalable approach to enhance public health monitoring systems.

## Methods

### Public health data

This study analyzed the drug prescription information dataset of Korea from 2002 to 2023, which was retrieved from the Public Data Portal website operated by the Ministry of Interior and Safety of Korea [22]. The drug prescription information dataset consists of a randomly selected sample of one million individuals from the NHIS beneficiaries who have received at least one prescription during each study year. The dataset provides detailed prescription information and basic demographic information, while removing several variable to prevent identification. We also retrieved sex, age and region-specific population size data from the Korean Stasistical Information Service (KOSIS).

We categorized age data into four age groups with 20-year interval based on previously documented age-related risk patterns and to ensure adequate sample sizes for analysis: 0–19, 20–39, 40–59 and over 60 (60 +). We consolidated regional data into seven major regions based on geographic proximity and historical disease patterns: Seoul (Seoul-si), Gyeonggi region (Incheon-si and Gyeonggi-do), Gangwon region (Gangwon-do), Chungcheong region (Daejeon-si, Sejong-si, Chungcheongnam-do, and Chungcheongbuk-do), - Jeolla region (Gwangju-si, Jeollabuk-do, and Jeollanam-do), Gyeongsang region (Daegu-si, Ulsan-si, Busan-si, Gyeongsangbuk-do, and Gyeongsangnam-do) and Jeju region (Jeju-do). Additional table and geographical map show this in detail [see Additional file 1].

### Strategies to identify clonorchiasis treatments records

Initially, we extracted all praziquantel prescription records from the dataset by using the ingredient code for praziquantel in Korea (216702ATB). We used the first 6 digits of ingredient code for dataset provided after 2022 since policy for the dataset has changed from providing full ingredient code to providing only partial codes to prevent identification. Then, we selected possible clonorchiasis cases by reviewing the dosing pattern. The recommended regimen for treatment of clonorchiasis in Korea is a dose of 10 to 25 mg/kg, three times daily, and a total dosage duration of a single day [23]. Based on this recommended treatment regimen, records with a total dosage duration of a single day, a daily dosage frequency of 3 times, and a dosage amount of 1 to 3 tablets (600 mg/tablet) were assumed to be clonorchiasis cases and selected for further analysis.

### Estimating the incidence of clonorchiasis

First, we checked whether recipients received multiple prescriptions of praziquantel in the same year. Based on the reference year and personal ID variable, we identified records under the same ID in the same year. Then, we reviewed the difference in date of care initiation to determine whether two different records correspond to re-infection of *C. sinensis* or not. We considered individuals who have prescription records with differences of at least 6 months as re-infection cases. We retained only the earliest record for individuals who have multiple prescription records which interval is less than 6 months to prevent overestimation.

We calculated the annual incidence rate of clonorchiasis in South Korea by dividing the number of estimated clonorchiasis cases with the number of sample population each year. Their 95% confidence intervals calculation based on Poisson exact method for rates were performed using *R* package ‘*Epitools*’. We also calculated sex, age group, and region-specific annual incidence rates using extracted variable-specific clonorchiasis case numbers and numbers of each sub-group within the sample population. Finally, we consolidated data from the first 5 years (2002–2006) and the final 5 years (2019–2023) and calculated region-specific incidences rates to evaluate notable temporal changes across regions. All data pre-processing, filtering, statistical analysis and visualizations were performed using *R* (version 4.4.1; R Core Team, 2024).

## Results

### Estimated clonorchiasis cases from prescription records

**Fig. 1:**
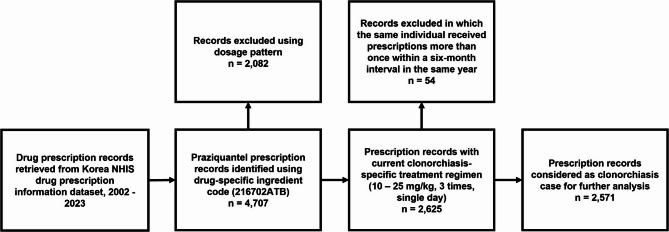
Study overview of eligibility assessment and screening

We initially identified 4,707 praziquantel prescription records from the dataset. (Fig. 1, Table 1) The most common prescription pattern, with 2,625 cases involved administering medication of three daily doses over one day, which is consistent with recommended clonorchiasis treatment regimen. This was followed by single daily doses (*n* = 408) commonly used for the treatment of intestinal flukes, and a three daily doses over two days (*n* = 583) regimen frequently prescribed for paragonimiasis, both of which were more prevalent compared to other patterns. Fifty-four additional records from individuals who were prescribed praziquantel more than once in a year has been excluded from records with clonorchiasis specific prescription pattern, thereby giving 2,571 clonorchiasis cases for further analysis.

**Table 1:** Prescription pattern of praziquantel prescription records

	Total dosage duration (day)^*^		
Daily dosage frequency (times/day)^*^	1	2	3
1	665	119	17
2	173	64	7
3	2710	604	62

^*^ For additional data with total dosage duration or daily dosage frequency exceeding 4, [see Additional file 1]

### Trends in the time-series incidence of clonorchiasis in Korea

**Fig. 2:**
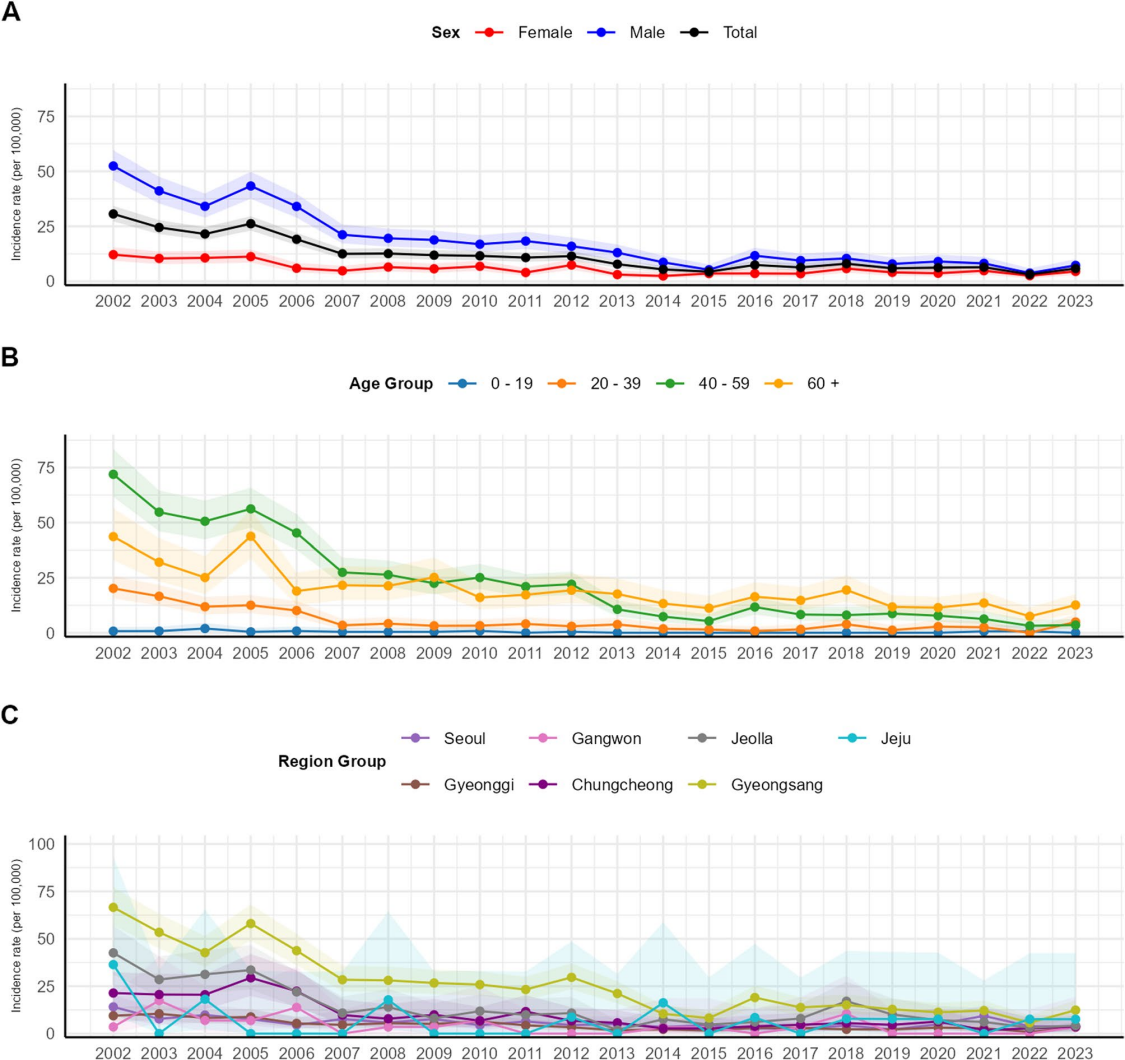
Estimated annual Sex-, age- and region- specific clonorchiasis incidence rate in South Korea (**A**) Annual clonorchiasis incidence rate and their respective 95% CI by sex. (**B**) Annual clonorchiasis incidence rate and their respective 95% CI by age group. (**C**) Annual clonorchiasis incidence rate and their respective 95% CI by major regions

Next, we examined the trend of estimated clonorchiasis incidence rate each year and the differences by sex. (Fig. 2A) From 2002 onward, the trend of overall incidence rate exhibited a general trend of continuous decline over time, from 30.63 (95% CI, 27.25–34.30) in 2002 to 5.80 (95% CI, 4.40–7.50) in 2023. When comparing incidence rates by sex, men consistently displayed a higher incidence rate across all intervals compared to women. Although the overall incidence rate and sex-specific incidence rates showed similar trends, some exceptions were observed. For instance, between 2004 and 2005, while the overall incidence rate increased from 21.52 (95% CI, 18.71–24.63) to 26.19 (95% CI, 23.09–29.59), the incidence rate among women changed from 10.64 (95% CI, 8.04–13.82) to 11.21 (95% CI, 8.53–14.46) while the incidence rate of men increased from 34.11 (95% CI, 28.95–39.92) to 43.35 (95% CI, 37.54–49.81). Conversely, between 2011 and 2012, when the incidence rate among men decreased from 18.29 (95% CI, 14.65–22.57) to 15.94 (95% CI, 12.56–19.95), the rate among women increased from 3.99 (95% CI, 2.47–6.10) to 7.39 (95% CI, 5.25–10.10).

We analyzed the trend in estimated clonorchiasis incidence by age group. (Fig. 2B) Similar to the overall incidence trend, the incidence rate for each age group showed a continuous decline over the 20-year period; however, there were distinct variations across age groups. The incidence rates were highest among the 40–59 age group and those aged 60 and older. Until 2012, the 40–59 age group exhibited the highest incidence rate with value from 71.94 (95% CI, 61.70–83.38) to 22.06 (95% CI, 17.23–27.83), but this trend reversed after 2013, with the 60 and older group consistently showing a higher incidence rate of 17.61 (95% CI, 12.12–24.73) in 2013 to 12.60 (95% CI, 8.78–17.53) in 2023. The 20–39 age group had incidence rate of 20.17 (95% CI, 15.53–25.76) in 2002, but this steadily decreased, stabilizing at a lower level after 2007, with incidence rates less than 5, reaching 4.88 (95% CI, 2.52–8.53) in 2023. The 0–19 age group exhibited an incidence rate of 0.70 (95% CI, 0.09–2.55) in 2002, and the rate remained consistently low thereafter. Since 2013, no cases have been reported, with the exception of a recorded incidence rate of 0.60 (95% CI, 0.02–3.40) in 2021 and 0.60 (95% CI, 0.02–3.37) in 2022.

We examined the trend in estimated clonorchiasis incidence by major regions each year. (Fig. 2C) Incidence trends of every region showed a general declining trend, as with the overall incidence trend. the Gyeongsang region consistently exhibited the highest incidence rate in most intervals, with rates of 66.58 (95% CI, 57.05–77.24) in 2002, 29.67 (95% CI, 23.41–37.08) in 2012 and 12.39 (95% CI, 8.36–17.68) in 2023. This was followed by Jeolla province, with rates of 42.57 (95% CI, 31.05–56.97) in 2002, 10.82 (95% CI, 5.40–19.36) in 2012 and 4.19 (95% CI, 1.14–10.73) in 2023.

### Incidence rate of first and last 5 years

**Fig. 3:**
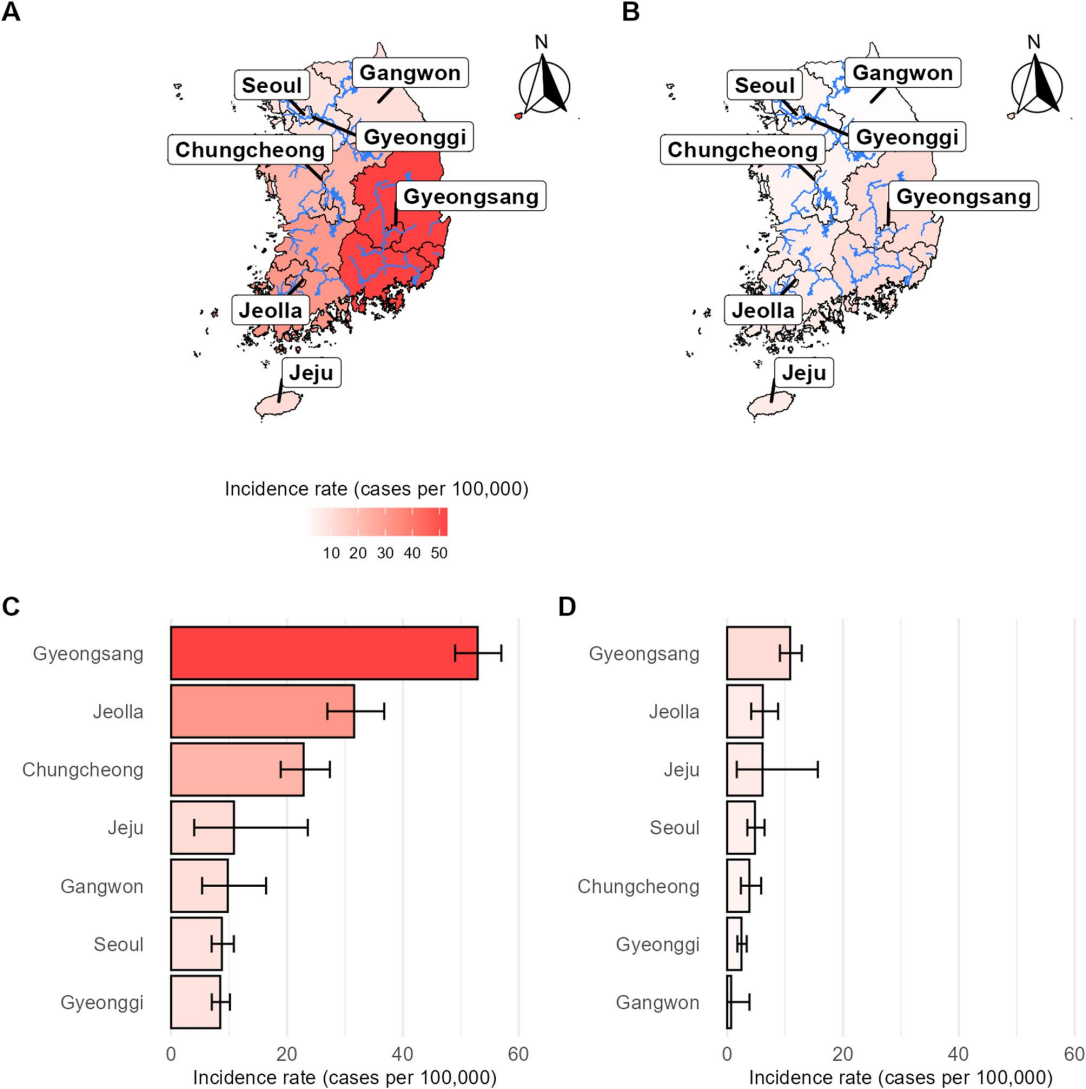
Estimated clonorchiasis incidence rate for the first and last 5 years of research period by region group (**A**) Visualized map of clonorchiasis incidence rate, calculated by aggregating data of first five years (2002–2006). (**B**) Visualized map of clonorchiasis incidence rate, calculated by aggregating data of last five years (2019–2023). (**C**) Bar plot and respective 95% CI of clonorchiasis incidence rate, calculated by aggregating data of first five years (2002–2006) (**D**) Bar plot and respective 95% CI of clonorchiasis incidence rate, calculated by aggregating data of last five years (2019–2023)

Finally, we compared the trend of estimated clonorchiasis incidence rates for the first five years (2002–2006) and last 5 years (2019–20223) of research period by major regions. (Fig. 3) Between the first five years, Gyeongsang Province exhibited the highest incidence rate at 52.90 (95% CI, 49.03–57.01), followed by Jeolla Province at 31.59 (95% CI, 26.97–36.78) and Chungcheong Province at 22.86 (95% CI, 18.91–27.40). Seventeen years later, during the period from 2019 to 2023, Gyeongsang and Jeolla Provinces retained the first and second highest incidence rates, respectively, but both saw a decrease of approximately 80%, achieving rates of 10.88 (95% CI: 9.12–12.89) and 6.16 (95% CI: 4.16–8.80). Chungcheong Province, previously ranked third, showed an even greater reduction of 83%, with its incidence rate dropping to 3.85 (95% CI: 2.38–5.88), ranking fifth among seven regions during the last 5 years. In contrast, Jeju Province, which had an incidence rate of 10.84 (95% CI: 3.98–23.59) and ranked fourth during the first five years, experienced a relatively modest decrease of 44% over the same period. By 2019–2023, Jeju’s incidence rate had decreased to 6.11 (95% CI: 1.67–15.65), making it the third highest among the regions.

## Discussion

Our analysis, utilizing the prescription datasets released from 2002 to 2023, enabled us to estimate the annual incidence rate of clonorchiasis in South Korea. The incidence rate of clonorchiasis in South Korea exhibited a generally consistent downward trend over the 22-year period. Additionally, a more detailed analysis based on sex, age, and regional variables indicated a higher incidence in men than in women, a higher incidence in age of 40 or more, and a tendency for higher incidence rates in the Gyeongsang, Jeolla, and Chungcheong regions.

The incidence of clonorchiasis estimated in this study was compared with various existing statistics collected in South Korea. Currently, the following types of data are gathered and managed in the country: sentinel surveillance statistics on clonorchiasis provided by the KDCA, National Survey of the Prevalence of Intestinal Parasites conducted intermittently by the KDCA, community-based surveys in endemic regions by the KDCA, and medical checkup results provided by the KAHP [11–17, 24]. Among these, the sentinel surveillance data—despite being compiled weekly in terms of case counts—were excluded from the comparison because the list of participating institutions is not publicly disclosed, their selection is not systematic, and is subject to arbitrary changes. Consequently, it is difficult to specify the source population for the data, which creates a fundamental problem with representativeness. Another consideration when making comparisons is that the remaining three sources of data all estimate prevalence based on egg-positive rates from stool examinations. Although a direct comparison between egg-positive rates and the incidence rates estimated in our study is not appropriate—since prevalence is not directly equivalent to incidence—the two measures are not entirely independent. Clonorchiasis can persist for years or even decades without treatment, so prevalence reflects the accumulation of past and current incidence. While factors such as early detection and treatment influence prevalence, in a setting where incidence continues to decline, prevalence is also expected to decrease over time. Therefore, prevalence will eventually follow the temporal trend of incidence rates.

From a population-wide perspective, both the existing prevalence statistics and our incidence estimates exhibit a consistent downward trend over time.(Supplementary material, Figure S2) Another point of convergence between our findings and established statistics is the disease’s persistence at a stable level, rather than its complete eradication. However, one unexpected finding in comparison is likely attributable to the nationwide survey itself. The estimated incidence rate of clonorchiasis in 2005 increased unexpectedly compared to 2004, deviating from the observed declining trend. (Fig. 2A) This outcome is presumed to have been influenced by the seventh nationwide survey conducted in 2004 and the subsequent policy measures [11]. Following the survey, individuals who tested positive for eggs received treatment, which may have led to the recording of newly identified positive cases from the survey as additional incidences in our estimation. Notably, the more pronounced increase in 2005 among men, individuals aged 40 and above, and populations in the Gyeongsang, Jeolla, and Chungcheong regions, corresponds to higher positivity rates observed in these groups during the survey [11]. This sudden increase in estimated incidence is likely attributable to the nationwide survey, further supporting the notion that our estimation reflects real-world data.

In terms of sex-specific statistics, the National Survey of the Prevalence of Intestinal Parasites reported egg-positive rates of 3.21% for men and 1.62% for women in 2004, and 2.47% for men and 1.25% for women in 2012 [11, 12]. Similarly, data from both the KAHP and endemic area surveys consistently showed that the prevalence among men was twice as high—or higher—than that among women across all periods [14–17, 24]. Our estimates of the incidence of clonorchiasis also demonstrated that the incidence rate among men was at least twice that of women each year. Given that sustained annual incidence trends would naturally converge toward corresponding prevalence levels over time, it is reasonable to infer that our incidence estimates exhibit a sex-specific pattern consistent with those observed in existing prevalence statistics. These patterns may be attributed to dietary behaviors in South Korea, where men have engaged more frequently in social activities involving alcohol consumption and consuming raw freshwater fish, compared to women [5, 6, 25]. 

From an age-based perspective, our estimated incidence rates show patterns that closely align with existing prevalence data. Among individuals under the age of 40, particularly those aged 0 to 19, our study estimated exceptionally low incidence rates, with no recorded cases in most years since 2013. Similarly, in endemic area surveys, which provide the most comparable data, this age group consistently exhibited markedly lower prevalence compared to older cohorts [14–17]. Given that these surveys are likely to overestimate prevalence relative to the general population, the alignment between our low incidence estimates and the low observed prevalence in this group supports the validity of our findings. For the 20–39 age group, a significant decline in egg-positive rates was observed over the past decade, which mirrors the sharp decrease in incidence observed in our study. Among individuals aged 40 and older, the KAHP data and endemic area surveys in 2011 showed that the 40–59 age group had a higher prevalence than those aged 60 and above [14–17, 24]. However, this gap gradually narrowed over time, and by 2020, the prevalence between the two groups had become nearly identical. Our incidence estimates show a corresponding shift: between 2002 and 2012, the highest incidence was observed in the 40–59 age group, but from 2013 onward, the incidence among individuals aged 60 and above surpassed that of the 40–59 age group. This convergence in prevalence between the two older age groups, as shown in existing data, is thus well-reflected in our incidence estimates, lending further credibility to the accuracy and reliability of our estimation. This shift may reflect the influence of a cohort effect on the decline in clonorchiasis prevalence [26]. Since the 1970 s, the South Korean government has implemented school-based health promotion initiatives, educating younger cohorts on the risks of consuming raw freshwater fish as a major transmission route for intestinal parasites [27]. Consequently, reductions in raw freshwater fish consumption among these younger cohorts may have led to a more pronounced decline in clonorchiasis prevalence within the 40–59 age group over time.

Regional comparisons between past prevalence statistics and our incidence estimates also reveal similar patterns. The KAHP data report egg-positive rates that encompass all parasitic infections, making it impossible to isolate the prevalence of clonorchiasis specifically. Nonetheless, KAHP statistics indicate that since the 2000 s, clonorchiasis has accounted for over 50% of all reported parasitic infections in South Korea [24]. Therefore, we were able to indirectly infer the egg-positive rate specific to *C. sinensis* by analyzing the overall egg-positive rates, allowing for a comparative assessment. The endemic area surveys, while focused on regions along the five major rivers in South Korea where clonorchiasis is most prevalent, aggregate data by river basin rather than by administrative divisions. Accordingly, when comparing our estimated incidence rates with the endemic area survey statistics, we based our evaluation on the regions through which the respective rivers flow. Within the KAHP data, the highest egg-positive rates were observed in areas we classified as part of Gyeongsang Province—specifically Gyeongsangnam-do, Ulsan, and Gyeongsangbuk-do—followed by Jeollanam-do, which we grouped under Jeolla Province [24]. Similarly, the endemic area surveys reported consistently high egg-positive rates in the Nakdong River basin (located in Gyeongsang Province), the Seomjin River basin (in Jeolla Province), and the Geum River basin (spanning both Jeolla and Chungcheong Provinces) [14–17]. These findings align well with our results, which showed the highest estimated incidence rates in Gyeongsang, followed by Jeolla and then Chungcheong. Taken together, this correspondence suggests that our incidence estimates are consistent with the spatial distribution patterns identified in previous prevalence surveys, despite differences in data structure and classification schemes. Notably, despite the Han River flowing through Gangwon Province, incidence rates in Gangwon Province are notably lower. This result may be attributed to the limited presence of freshwater fish infected with *C. sinensis* metacercariae in certain streams and the differing ecological distribution of the primary intermediate host (freshwater snails) in this region [28]. 

These findings underscore that the methodological approach utilized in this study can produce results comparable to those derived from existing clonorchiasis surveys or surveillance, while offering distinct advantages that make it a valuable complement to conventional systems. Direct surveys based on stool examination, for example, are cross-sectional by nature, providing prevalence only at the time of survey collection. Such surveys also demand significant time, human resources, and logistical effort. In addition, site selection is limited to endemic areas and is not based on general population demographics. Voluntary enrollment also limits the use of the collected data to estimate population-wide prevalence. Next, the sentinel surveillance system, with weekly reporting requirements, is run by the KDCA for several helminthiasis including clonorchiasis [19]. However, the number of sentinel institutions is limited, and the system only reports case counts, lacking the ability to provide demographic information on the infected population [29]. In contrast, utilizing data from the NHIS allows for a comprehensive population-level assessment and more robust aggregation of estimated clonorchiasis cases due to its electronic recording of prescription information. Additionally, since the health insurance data represents nationwide, real-world data, we were able to present risk maps of clonorchiasis based on this data without considering preferential sampling or data heterogeneity [30]. Finally, the approach of leveraging health insurance data may be extended to the surveillance of other diseases, particularly rare diseases for which dedicated surveillance systems are not feasible. This indirect method of disease monitoring offers promising applications for improving public health surveillance frameworks.

Our study has several possible limitations. First, the incidence of clonorchiasis may be underestimated. Infections can be asymptomatic or present with only mild symptoms for extended periods, meaning individuals may not seek diagnosis or treatment. However, quantitatively adjusting for this underestimation is not currently feasible, as it would require comprehensive data, such as symptom-based assessments with fecal examination screening programs, which is unavailable in Korea. Nonetheless, given that clonorchiasis is closely linked to dietary habits and adult worm can live for approximately 20 to 30 years, it is likely that the number of parasites carried by infected individuals accumulates over time. As a result, symptoms are expected to worsen, thereby increasing the likelihood of eventual detection. While diagnosis may be delayed, lifelong asymptomatic cases are likely rare.

Second, the potential for praziquantel treatment failure could affect the accuracy of our estimates. While a foundational study conducted in Korea reported an 85.7% cure rate for the standard regimen, and contemporary cure rates are likely higher due to lower average worm burdens, persistent infections requiring retreatment can lead to overestimation if a single case is counted multiple times [31]. To mitigate this risk, we analyzed our dataset for repeated prescriptions. We found that out of 2,625 total prescription records, 102 (3.9%) represented two or more prescriptions issued to the same individual (*n* = 48) within a six-month interval. Interpreting these instances as likely cases of treatment failure or unresolved infection (Fig. 1), we counted each patient only once within this timeframe to minimize overestimation in our incidence calculations.

Third, the prescription dataset, derived from NHIS beneficiaries who have received at least one prescription, may not be fully representative of the general population. To assess this, we compared the demographic composition of three groups: the national census population, NHIS beneficiaries who received prescriptions, and the prescription dataset used in this study. Our analysis confirmed that the NHIS beneficiaries prescription population (covering 80–90% of national population) showed nearly identical distributions across age (Supplementary Material, Table S9). Similarly, comparisons between the prescription dataset and the total population revealed closely aligned distributions across sex, age, and region. (Supplementary material, Figure S3, S4, S5) While we observed a slight overrepresentation of females and individuals in the 0–19 and 60 + age groups in the prescription datasets, these differences were not substantial. Therefore, we consider that sampling bias was minimal and the estimated incidence rates are generalizable to the entire Korean population.

Finally, our analysis assumes that all clonorchiasis patients are prescribed praziquantel according to the therapeutic regimen specific to clonorchiasis, which is distinct from the regimens used for other infections. This assumption introduces a potential for misclassification from two sources. First, incidence could be underestimated if clonorchiasis patients are prescribed a non-standard praziquantel regimen (a false negative). Conversely, incidence could be overestimated if non-clonorchiasis patients (e.g. paragonimiasis) are mistakenly prescribed the clonorchiasis-specific regimen (a false positive). While we argue the latter scenario is likely rare given the low prevalence of paragonimiasis treated with praziquantel in Korea, both possibilities represent an inherent limitation of a case definition based on prescription patterns.

## Conclusion

In this study, we propose a method to estimate the time-series trend of annual clonorchiasis incidence, using publicly available pharmaceutical prescription data. Our method leverages disease-specific prescription patterns to estimate annual sex-, age-, and region-specific clonorchiasis incidence rates. When compared to existing clonorchiasis statistics, our estimates generally showed consistent trends, indicating that this cost-effective approach is a suitable complementary tool for traditional disease surveillance systems or surveys. Future research can build on these findings to generate incidence statistics for rare diseases where the establishment of dedicated surveillance systems is challenging.

## Supplementary Information

Supplementary Material 1.

## Data Availability

The drug prescription information dataset analysed during the current study is available from the Public Data Portal website operated by the Ministry of Interior and Safety of Korea, https://www.data.go.kr/en/data/15007117/fileData.do.
