# Supplementary material for: Assessing trends in clonorchiasis incidence via prescription data analysis in South Korea
Source: BMC Public Health. 2025 Aug 21;25:2873. doi: 10.1186/s12889-025-23893-9 (PMC12369146; doi:10.1186/s12889-025-23893-9)
Supplement: Supplementary file 1 — Supplementary Material 1. [file 12889_2025_23893_MOESM1_ESM.docx]

**Supplementary Materials**

**Table of Contents**

**Supplementary Figure**

**Figure S1.** Visualization of South Korean map by assigned region groups

**Figure S2.** Time-series trend of existing clonorchiasis prevalence statistics and our incidence estimates

**Figure S3.** Comparison of NHIS beneficiaries who received prescription at least once and national census data by age group

**Figure S4.** Comparison of drug prescription information dataset and national population data by sex

**Figure S5.** Comparison of drug prescription information dataset and national population data by age group

**Figure S6.** Comparison of drug prescription information dataset and national population data by major regions

**Supplementary Tables**

**Table S1**. List of South Korean regions and its abbreviations used in the study

**Table S2**. Prescription pattern of praziquantel records extracted from the drug prescription information dataset, 2002 – 2023

**Table S3**. Estimated sex-specific and total clonorchiasis incidence rate in South Korea, 2002 – 2023

**Table S4**. Estimated age group-specific clonorchiasis incidence rate in South Korea, 2002 - 2023

**Table S5**. Estimated major region-specific clonorchiasis incidence rate in South Korea, 2002 – 2023

**Table S6.** Number of estimated raw clonorchiasis cases in the dataset by sex

**Table S7.** Number of estimated raw clonorchiasis cases in the dataset by age group

**Table S8.** Number of estimated raw clonorchiasis cases in the dataset by major regions

**Table S9.** Number of NHIS beneficiaries who received prescription at least once

Figure S1. Visualization of South Korean map by assigned region groups


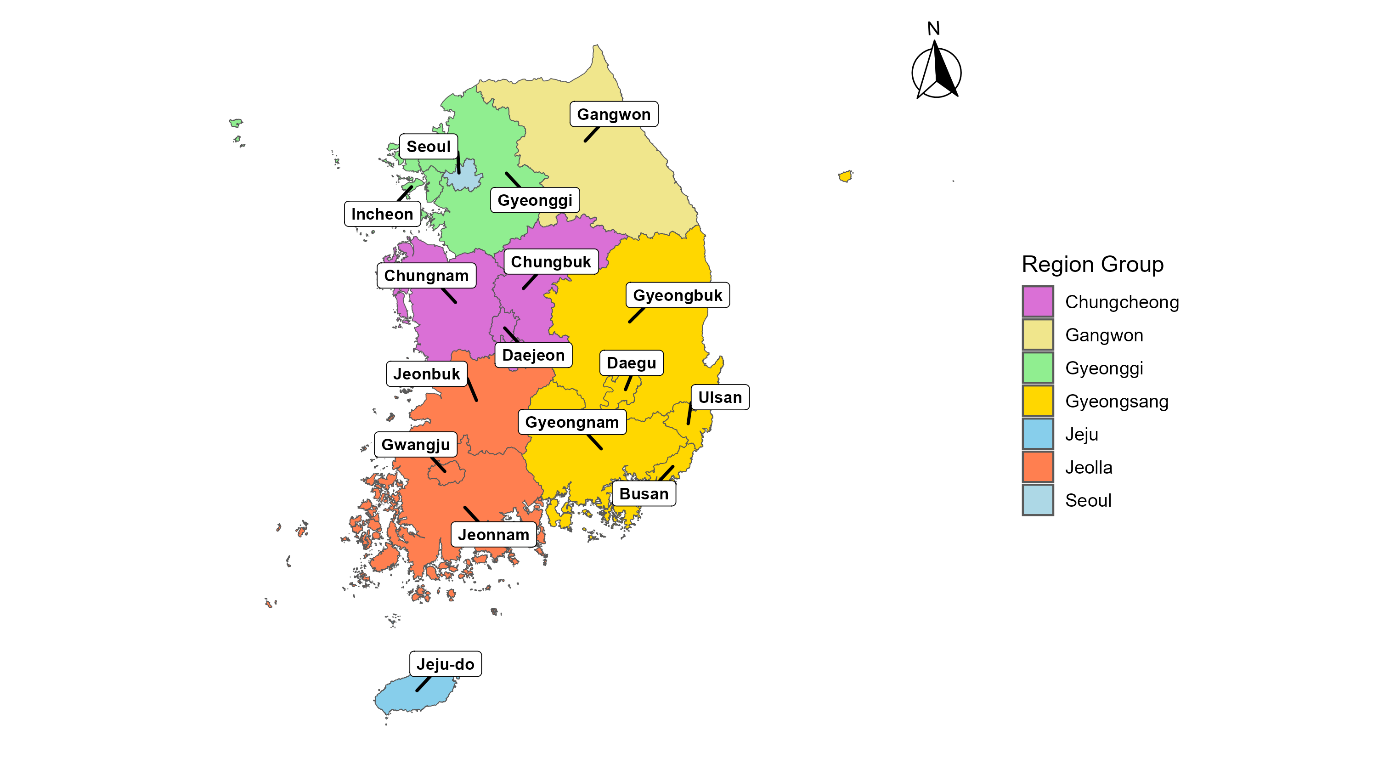


**Figure S2.** Time-series trend of existing clonorchiasis prevalence statistics and our incidence estimates


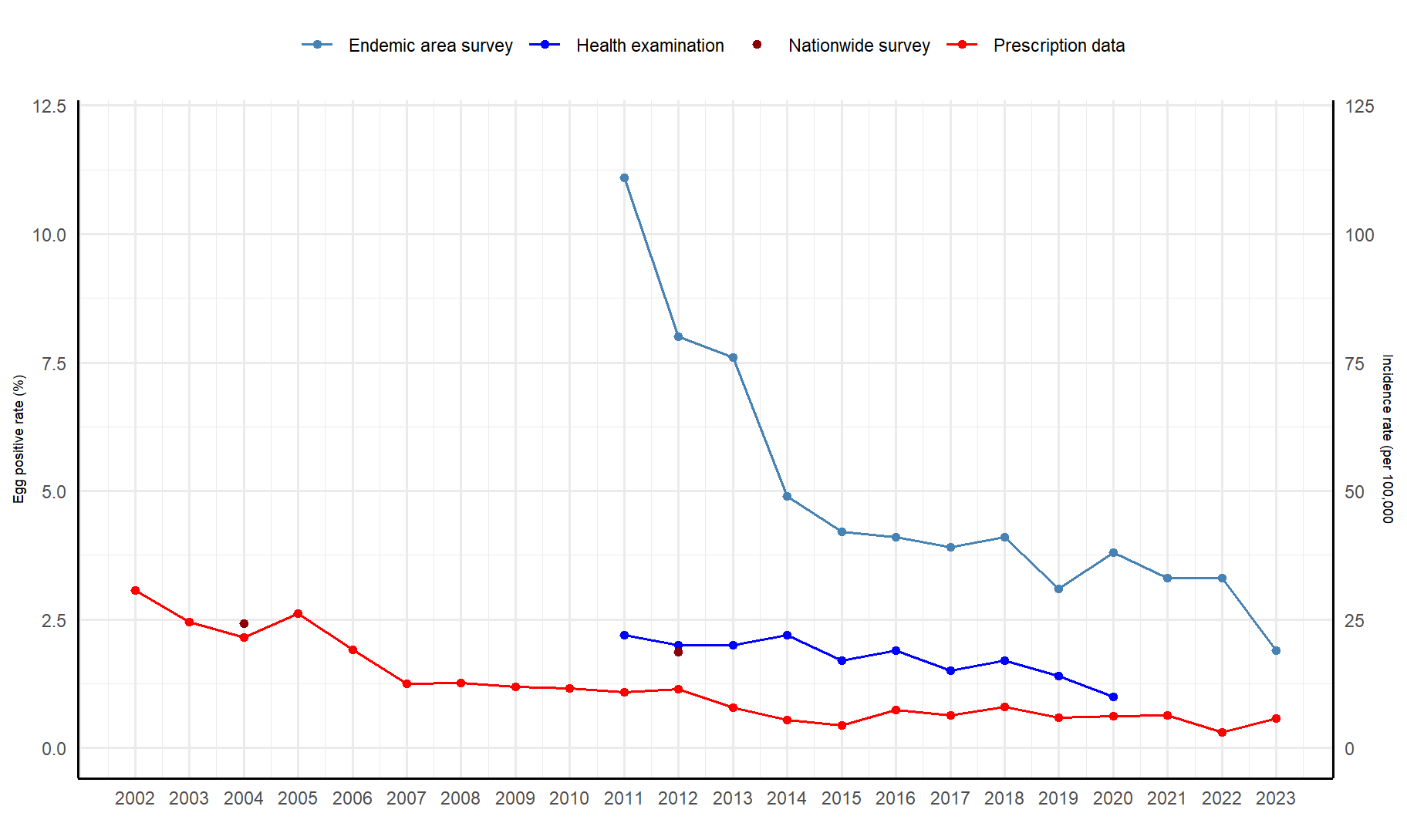


**Figure S3.** Comparison of NHIS beneficiaries who received prescription at least once and national census data by age group

**
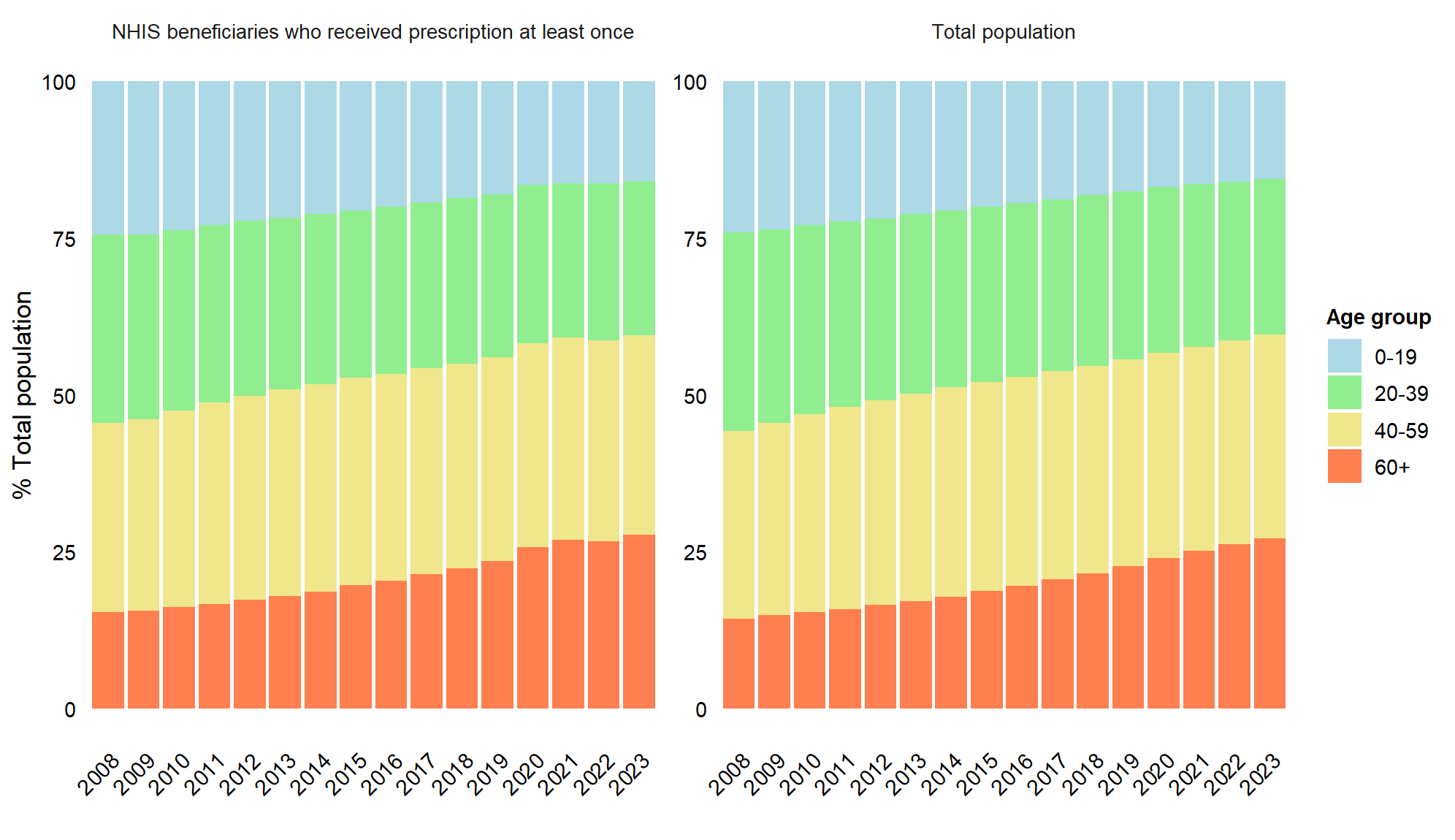
**

**Figure S4.** Comparison of drug prescription information dataset and national population data by sex


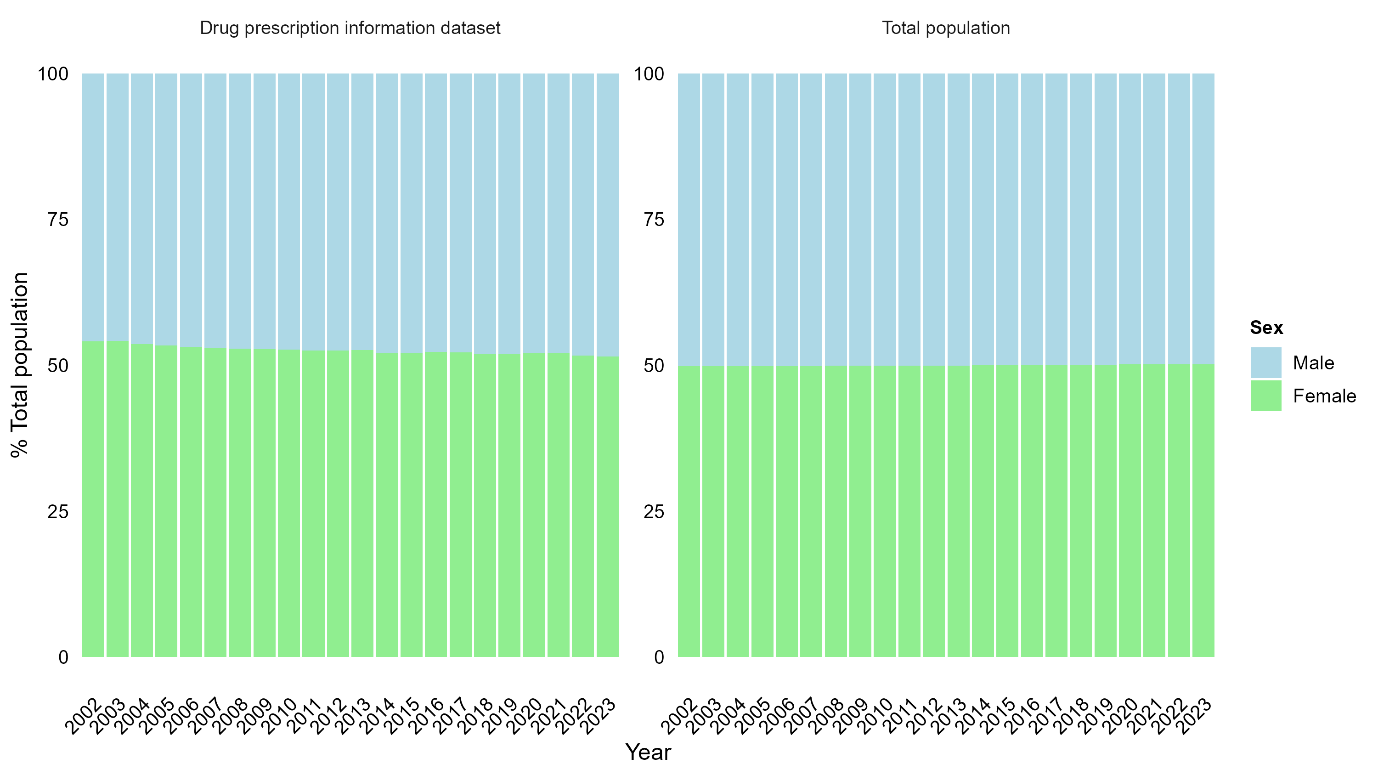


**Figure S5.** Comparison of drug prescription information dataset and national population data by age


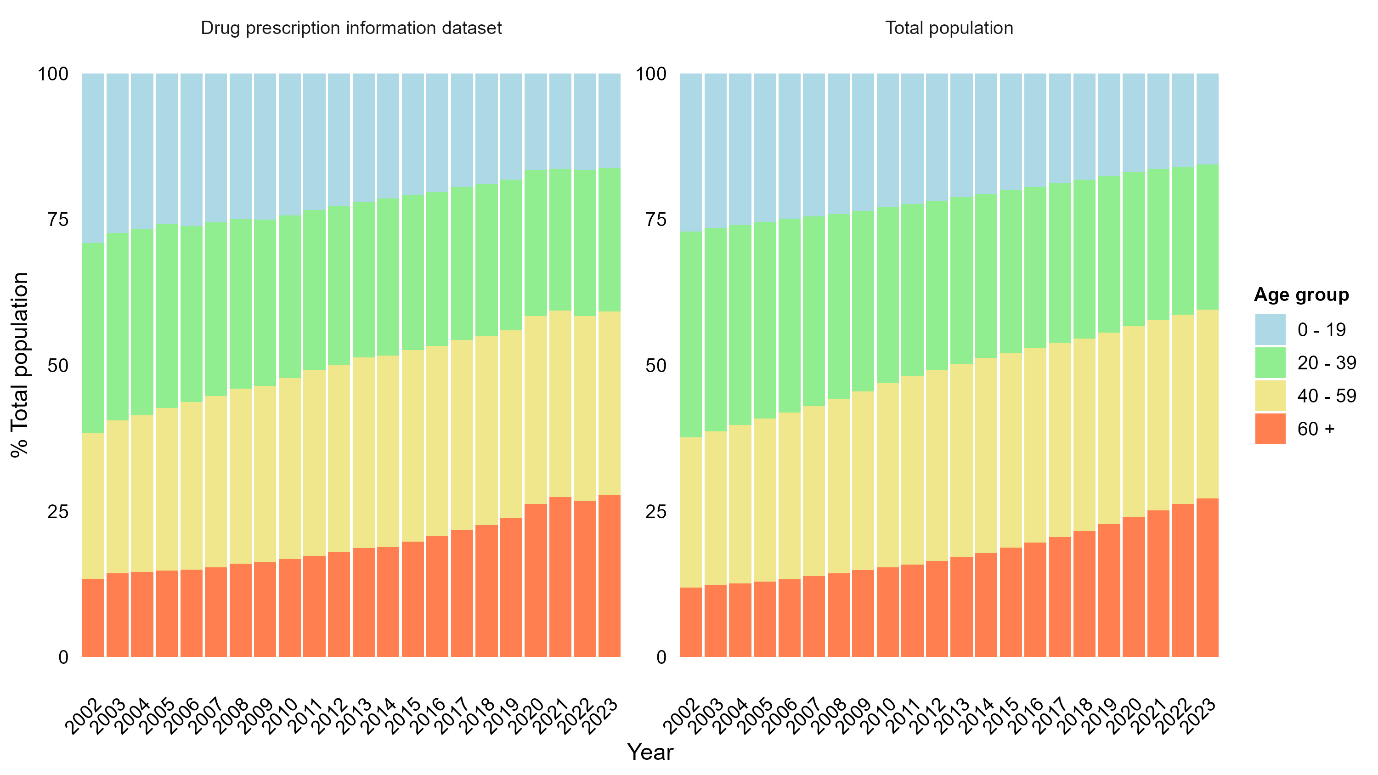


**Figure S6.** Comparison of drug prescription information dataset and national population data by major regions

**
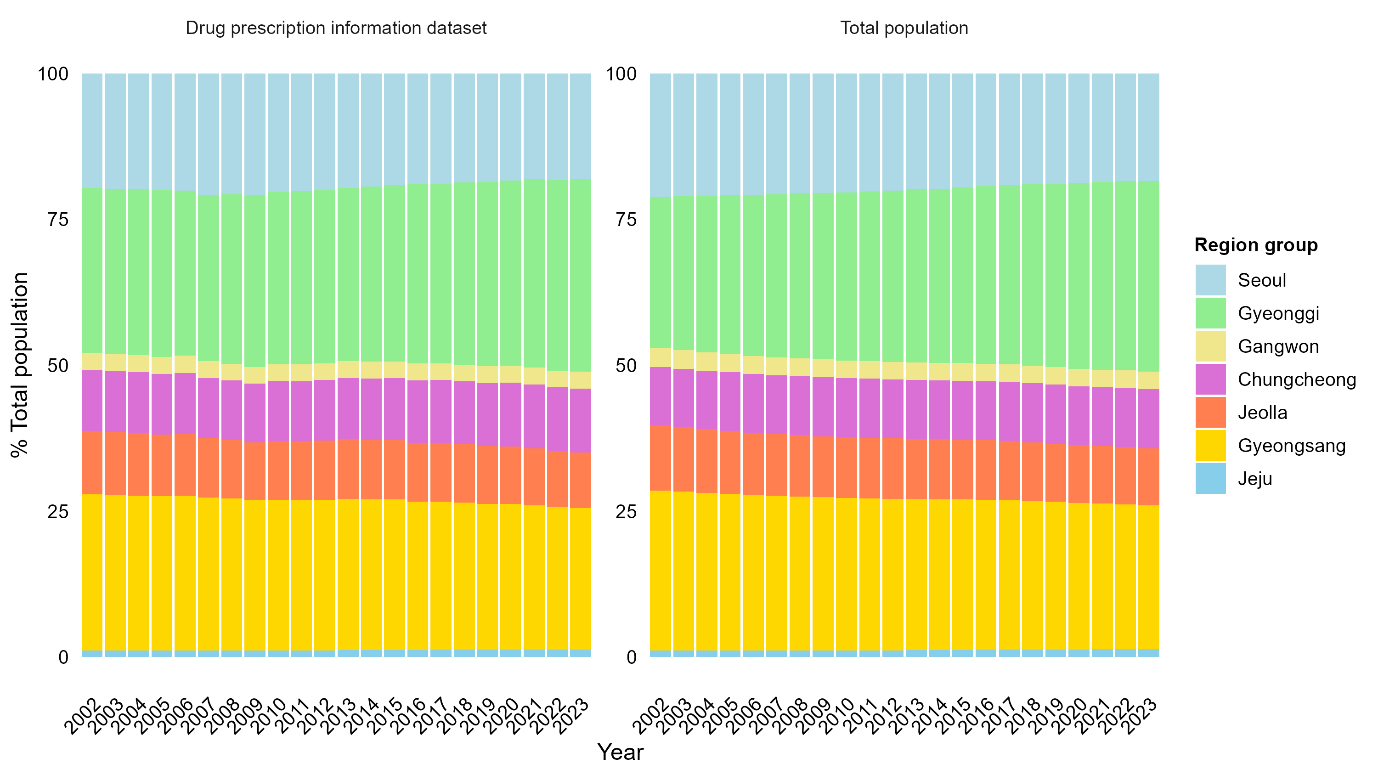
**

Table S1. List of South Korean regions and its abbreviations used in the study

| **Full region name** | **Abbreviated region name** |
| --- | --- |
| Seoul-si | Seoul |
| Busan-si | Busan |
| Daegu-si | Daegu |
| Incheon-si | Incheon |
| Gwangju-si | Gwangju |
| Daejeon-si | Daejeon |
| Ulsan-si | Ulsan |
| Gyeonggi-do | Gyeonggi |
| Gangwon-do | Gangwon |
| Chungcheongbuk-do | Chungbuk |
| Chungcheongnam-do | Chungnam |
| Jeollanam-do | Jeonnam |
| Jeollabuk-do | Jeonbuk |
| Gyeongsangbuk-do | Gyeongbuk |
| Gyeongsangnam-do | Gyeongnam |
| Jeju-do | Jeju-do |

Table S2. Prescription pattern of praziquantel records extracted from the drug prescription information dataset, 2002 – 2023

|  | Total dosage duration (day) | | | | | | | | | | | | | | | | | |
| --- | --- | --- | --- | --- | --- | --- | --- | --- | --- | --- | --- | --- | --- | --- | --- | --- | --- | --- |
| Daily dosage frequency  (times/day) | 1 | 2 | 3 | 4 | 5 | 6 | 7 | 8 | 9 | 10 | 12 | 14 | 15 | 16 | 24 | 28 | 30 | 60 |
| 0 | 408 | 91 | 13 | 3 | 7 | 2 | 5 | 20 | 0 | 408 | 91 | 13 | 3 | 7 | 2 | 5 | 20 | 0 |
| 1 | 139 | 55 | 5 | 11 | 0 | 0 | 7 | 1 | 1 | 139 | 55 | 5 | 11 | 0 | 0 | 7 | 1 | 1 |
| 2 | 2625 | 583 | 61 | 5 | 4 | 1 | 19 | 0 | 0 | 2625 | 583 | 61 | 5 | 4 | 1 | 19 | 0 | 0 |
| 3 | 123 | 22 | 1 | 1 | 0 | 0 | 1 | 0 | 0 | 123 | 22 | 1 | 1 | 0 | 0 | 1 | 0 | 0 |
| 4 | 2 | 0 | 0 | 0 | 0 | 0 | 0 | 0 | 0 | 2 | 0 | 0 | 0 | 0 | 0 | 0 | 0 | 0 |
| 6 | 1 | 0 | 0 | 0 | 0 | 0 | 0 | 0 | 0 | 1 | 0 | 0 | 0 | 0 | 0 | 0 | 0 | 0 |
| 7 | 3 | 0 | 0 | 0 | 0 | 0 | 0 | 0 | 0 | 3 | 0 | 0 | 0 | 0 | 0 | 0 | 0 | 0 |
| 8 | 0 | 1 | 0 | 0 | 0 | 0 | 0 | 0 | 0 | 0 | 1 | 0 | 0 | 0 | 0 | 0 | 0 | 0 |
| 9 | 408 | 91 | 13 | 3 | 7 | 2 | 5 | 20 | 0 | 408 | 91 | 13 | 3 | 7 | 2 | 5 | 20 | 0 |

Table S3. Estimated sex-specific and total clonorchiasis incidence rate in South Korea, 2002 - 2023

| Year | Male | Female | Total |
| --- | --- | --- | --- |
| 2002 | 52.45 (45.96 - 59.6) | 12.12 (9.33 - 15.47) | 30.63 (27.25 - 34.3) |
| 2003 | 41.12 (35.39 - 47.5) | 10.41 (7.84 - 13.55) | 24.49 (21.48 - 27.8) |
| 2004 | 34.11 (28.95 - 39.92) | 10.64 (8.04 - 13.82) | 21.52 (18.71 - 24.63) |
| 2005 | 43.35 (37.54 - 49.81) | 11.21 (8.54 - 14.46) | 26.19 (23.09 - 29.59) |
| 2006 | 34.04 (28.93 - 39.81) | 5.93 (4.03 - 8.41) | 19.1 (16.47 - 22.03) |
| 2007 | 21.17 (17.21 - 25.78) | 4.75 (3.07 - 7.01) | 12.48 (10.38 - 14.87) |
| 2008 | 19.55 (15.76 - 23.97) | 6.45 (4.46 - 9.01) | 12.62 (10.51 - 15.03) |
| 2009 | 18.8 (15.1 - 23.13) | 5.68 (3.83 - 8.1) | 11.88 (9.84 - 14.21) |
| 2010 | 16.86 (13.37 - 20.98) | 6.81 (4.77 - 9.43) | 11.57 (9.56 - 13.87) |
| 2011 | 18.29 (14.65 - 22.57) | 3.99 (2.47 - 6.1) | 10.78 (8.84 - 13.01) |
| 2012 | 15.94 (12.56 - 19.95) | 7.39 (5.25 - 10.1) | 11.45 (9.45 - 13.74) |
| 2013 | 13.02 (9.99 - 16.7) | 3.03 (1.73 - 4.92) | 7.77 (6.14 - 9.69) |
| 2014 | 8.64 (6.25 - 11.63) | 2.41 (1.28 - 4.11) | 5.39 (4.07 - 7) |
| 2015 | 5.23 (3.41 - 7.66) | 3.52 (2.12 - 5.5) | 4.34 (3.16 - 5.8) |
| 2016 | 11.65 (8.75 - 15.2) | 3.55 (2.1 - 5.61) | 7.42 (5.8 - 9.34) |
| 2017 | 9.41 (6.86 - 12.59) | 3.45 (2.04 - 5.45) | 6.3 (4.84 - 8.06) |
| 2018 | 10.41 (7.72 - 13.72) | 5.77 (3.9 - 8.24) | 8 (6.34 - 9.96) |
| 2019 | 7.91 (5.59 - 10.85) | 4.04 (2.5 - 6.18) | 5.9 (4.49 - 7.61) |
| 2020 | 8.98 (6.5 - 12.1) | 3.64 (2.19 - 5.69) | 6.2 (4.75 - 7.95) |
| 2021 | 8.14 (5.79 - 11.13) | 4.8 (3.11 - 7.08) | 6.4 (4.93 - 8.17) |
| 2022 | 3.72 (2.21 - 5.88) | 2.52 (1.34 - 4.31) | 3.1 (2.11 - 4.4) |
| 2023 | 7.22 (5.03 - 10.04) | 4.46 (2.83 - 6.7) | 5.8 (4.4 - 7.5) |

Table S4. Estimated age group-specific clonorchiasis incidence rate in South Korea, 2002 - 2023

|  | Age groups (20-year) | | | |
| --- | --- | --- | --- | --- |
| Year | 0 - 19 | 20 - 39 | 40 - 59 | 60 + |
| 2002 | 0.71 (0.09 - 2.55) | 20.17 (15.53 - 25.76) | 71.94 (61.7 - 83.38) | 43.61 (33.03 - 56.5) |
| 2003 | 0.75 (0.09 - 2.71) | 16.59 (12.39 - 21.75) | 54.73 (46.04 - 64.58) | 32.07 (23.39 - 42.92) |
| 2004 | 1.91 (0.62 - 4.46) | 11.84 (8.33 - 16.31) | 50.58 (42.35 - 59.94) | 25.11 (17.59 - 34.76) |
| 2005 | 0.39 (0.01 - 2.2) | 12.52 (8.9 - 17.12) | 56.21 (47.69 - 65.83) | 43.76 (33.7 - 55.88) |
| 2006 | 0.78 (0.09 - 2.81) | 10.1 (6.82 - 14.42) | 45.3 (37.79 - 53.86) | 18.95 (12.59 - 27.38) |
| 2007 | 0.39 (0.01 - 2.19) | 3.38 (1.62 - 6.22) | 27.44 (21.76 - 34.15) | 21.6 (14.87 - 30.33) |
| 2008 | 0.4 (0.01 - 2.24) | 4.13 (2.14 - 7.22) | 26.38 (20.89 - 32.88) | 21.34 (14.78 - 29.82) |
| 2009 | 0.4 (0.01 - 2.22) | 3.15 (1.44 - 5.97) | 22.49 (17.47 - 28.51) | 25.18 (18.07 - 34.16) |
| 2010 | 0.82 (0.1 - 2.96) | 3.21 (1.47 - 6.1) | 25.1 (19.84 - 31.33) | 16.04 (10.57 - 23.34) |
| 2011 | 0 (0 - 1.57) | 4 (2 - 7.16) | 21 (16.27 - 26.67) | 17.27 (11.65 - 24.65) |
| 2012 | 0.44 (0.01 - 2.43) | 2.93 (1.27 - 5.78) | 22.06 (17.23 - 27.83) | 19.33 (13.46 - 26.88) |
| 2013 | 0 (0 - 1.66) | 3.75 (1.8 - 6.9) | 10.65 (7.42 - 14.81) | 17.61 (12.12 - 24.73) |
| 2014 | 0 (0 - 1.66) | 1.79 (0.58 - 4.18) | 7.33 (4.75 - 10.82) | 13.29 (8.68 - 19.47) |
| 2015 | 0 (0 - 1.7) | 1.45 (0.4 - 3.72) | 5.3 (3.14 - 8.37) | 11.19 (7.09 - 16.79) |
| 2016 | 0 (0 - 1.87) | 0.78 (0.09 - 2.83) | 11.7 (8.24 - 16.13) | 16.38 (11.27 - 23) |
| 2017 | 0 (0 - 1.9) | 1.52 (0.42 - 3.9) | 8.29 (5.46 - 12.06) | 14.72 (10.07 - 20.78) |
| 2018 | 0 (0 - 1.95) | 3.83 (1.83 - 7.04) | 8.04 (5.25 - 11.78) | 19.44 (14.12 - 26.09) |
| 2019 | 0 (0 - 2.02) | 1.16 (0.24 - 3.4) | 8.71 (5.79 - 12.59) | 11.76 (7.81 - 16.99) |
| 2020 | 0 (0 - 2.23) | 2.8 (1.12 - 5.76) | 7.76 (5.02 - 11.46) | 11.43 (7.71 - 16.32) |
| 2021 | 0.61 (0.02 - 3.4) | 2.48 (0.91 - 5.39) | 6.26 (3.82 - 9.66) | 13.51 (9.51 - 18.62) |
| 2022 | 0.61 (0.02 - 3.37) | 0 (0 - 1.47) | 3.16 (1.52 - 5.81) | 7.47 (4.56 - 11.53) |
| 2023 | 0 (0 - 2.28) | 4.89 (2.52 - 8.54) | 3.49 (1.74 - 6.25) | 12.61 (8.78 - 17.53) |

Table S5. Estimated major region-specific clonorchiasis incidence rate in South Korea, 2002 - 2023

|  | Major regions | | | | | | |
| --- | --- | --- | --- | --- | --- | --- | --- |
| Year | Seoul | Gyeonggi | Gangwon | Chungcheong | Jeolla | Gyeongsang | Jeju |
| 2002 | 14.09 (9.29 - 20.5) | 9.43 (6.16 - 13.82) | 3.56 (0.09 - 19.82) | 21.41 (13.42 - 32.41) | 42.57 (31.05 - 56.97) | 66.58 (57.05 - 77.24) | 36.39 (9.92 - 93.18) |
| 2003 | 7.78 (4.36 - 12.84) | 10.49 (7.03 - 15.07) | 17.45 (5.67 - 40.73) | 20.62 (12.76 - 31.52) | 28.51 (19.24 - 40.7) | 53.42 (44.91 - 63.07) | 0 (0 - 33.71) |
| 2004 | 9.82 (5.91 - 15.33) | 8.23 (5.22 - 12.35) | 6.98 (0.85 - 25.21) | 20.52 (12.7 - 31.36) | 31.24 (21.51 - 43.88) | 42.7 (35.13 - 51.42) | 18.13 (2.2 - 65.51) |
| 2005 | 7.65 (4.28 - 12.62) | 8.85 (5.72 - 13.06) | 6.92 (0.84 - 25.01) | 29.41 (19.85 - 41.99) | 33.54 (23.36 - 46.65) | 58.02 (49.14 - 68.05) | 0 (0 - 33.36) |
| 2006 | 4.56 (2.08 - 8.65) | 5.39 (3.01 - 8.88) | 13.79 (3.76 - 35.3) | 22.38 (14.19 - 33.58) | 21.99 (13.94 - 32.99) | 43.74 (36.08 - 52.55) | 0 (0 - 32.52) |
| 2007 | 7.71 (4.41 - 12.52) | 4.6 (2.45 - 7.87) | 0 (0 - 12.7) | 9.81 (4.7 - 18.03) | 10.86 (5.42 - 19.44) | 28.42 (22.32 - 35.68) | 0 (0 - 32.76) |
| 2008 | 5.8 (3 - 10.13) | 5.52 (3.16 - 8.97) | 3.5 (0.09 - 19.52) | 7.85 (3.39 - 15.48) | 14.01 (7.66 - 23.5) | 28.07 (22 - 35.3) | 17.8 (2.16 - 64.31) |
| 2009 | 7.68 (4.39 - 12.47) | 5.07 (2.84 - 8.36) | 3.53 (0.09 - 19.64) | 9.91 (4.75 - 18.22) | 8.13 (3.51 - 16.02) | 26.69 (20.77 - 33.78) | 0 (0 - 32.83) |
| 2010 | 4.41 (2.02 - 8.38) | 6.43 (3.87 - 10.03) | 6.91 (0.84 - 24.98) | 6.8 (2.73 - 14.01) | 11.84 (6.12 - 20.69) | 25.87 (20.05 - 32.86) | 0 (0 - 33.18) |
| 2011 | 6.43 (3.42 - 11) | 4.37 (2.33 - 7.47) | 0 (0 - 12.78) | 11.63 (6.01 - 20.31) | 9.9 (4.75 - 18.21) | 23.25 (17.74 - 29.93) | 0 (0 - 32.69) |
| 2012 | 4.49 (2.05 - 8.53) | 3.35 (1.61 - 6.16) | 0 (0 - 12.71) | 6.73 (2.7 - 13.86) | 10.82 (5.4 - 19.36) | 29.67 (23.41 - 37.08) | 8.72 (0.22 - 48.58) |
| 2013 | 5.09 (2.44 - 9.35) | 1.68 (0.54 - 3.91) | 0 (0 - 12.59) | 5.7 (2.09 - 12.41) | 1.94 (0.23 - 7) | 21.14 (15.93 - 27.52) | 0 (0 - 31.57) |
| 2014 | 3.48 (1.4 - 7.17) | 2.25 (0.9 - 4.63) | 3.33 (0.08 - 18.56) | 2.75 (0.57 - 8.04) | 7.57 (3.27 - 14.91) | 10.43 (6.93 - 15.08) | 16.27 (1.97 - 58.77) |
| 2015 | 4.53 (2.07 - 8.59) | 1.6 (0.52 - 3.73) | 3.33 (0.08 - 18.55) | 2.72 (0.56 - 7.96) | 4.75 (1.54 - 11.08) | 8.22 (5.15 - 12.45) | 0 (0 - 29.67) |
| 2016 | 2.71 (0.88 - 6.33) | 3.02 (1.38 - 5.73) | 0 (0 - 13.11) | 3.86 (1.05 - 9.89) | 6.14 (2.25 - 13.37) | 19.03 (13.98 - 25.3) | 8.48 (0.21 - 47.23) |
| 2017 | 2.64 (0.86 - 6.16) | 2.93 (1.34 - 5.56) | 3.47 (0.09 - 19.34) | 4.65 (1.51 - 10.86) | 7.96 (3.44 - 15.68) | 13.78 (9.6 - 19.17) | 0 (0 - 29.54) |
| 2018 | 4.29 (1.85 - 8.45) | 2.24 (0.9 - 4.62) | 10.44 (2.15 - 30.5) | 5.55 (2.04 - 12.08) | 17.07 (9.94 - 27.33) | 15.08 (10.67 - 20.7) | 7.82 (0.2 - 43.59) |
| 2019 | 2.15 (0.59 - 5.5) | 2.22 (0.89 - 4.57) | 0 (0 - 12.84) | 4.62 (1.5 - 10.78) | 10.13 (4.86 - 18.63) | 12.82 (8.77 - 18.1) | 7.72 (0.2 - 43.02) |
| 2020 | 4.91 (2.24 - 9.32) | 3.15 (1.51 - 5.8) | 0 (0 - 12.77) | 6.38 (2.57 - 13.15) | 7.08 (2.85 - 14.59) | 11.25 (7.47 - 16.25) | 7.67 (0.19 - 42.73) |
| 2021 | 9.38 (5.46 - 15.02) | 2.79 (1.28 - 5.3) | 0 (0 - 12.65) | 1.83 (0.22 - 6.61) | 6.16 (2.26 - 13.4) | 12.14 (8.19 - 17.33) | 0 (0 - 28.07) |
| 2022 | 3.83 (1.54 - 7.9) | 0.92 (0.19 - 2.69) | 0 (0 - 12.87) | 2.75 (0.57 - 8.03) | 3.11 (0.64 - 9.1) | 5.75 (3.14 - 9.65) | 7.61 (0.19 - 42.39) |
| 2023 | 3.86 (1.55 - 7.95) | 3.34 (1.67 - 5.97) | 3.47 (0.09 - 19.32) | 3.66 (1 - 9.37) | 4.19 (1.14 - 10.73) | 12.39 (8.36 - 17.68) | 7.61 (0.19 - 42.38) |

**Table S6.** Number of estimated raw clonorchiasis cases in the dataset by sex

|  | Sex | |
| --- | --- | --- |
| Year | Male | Female |
| 2002 | 235 | 64 |
| 2003 | 184 | 55 |
| 2004 | 155 | 56 |
| 2005 | 199 | 59 |
| 2006 | 157 | 31 |
| 2007 | 99 | 25 |
| 2008 | 92 | 34 |
| 2009 | 89 | 30 |
| 2010 | 80 | 36 |
| 2011 | 87 | 21 |
| 2012 | 76 | 39 |
| 2013 | 62 | 16 |
| 2014 | 43 | 13 |
| 2015 | 26 | 19 |
| 2016 | 54 | 18 |
| 2017 | 45 | 18 |
| 2018 | 50 | 30 |
| 2019 | 38 | 21 |
| 2020 | 43 | 19 |
| 2021 | 39 | 25 |
| 2022 | 18 | 13 |
| 2023 | 35 | 23 |

**Table S7.** Number of estimated raw clonorchiasis cases in the dataset by age group

|  | Age group | | | |
| --- | --- | --- | --- | --- |
| Year | 0 - 19 | 20 - 39 | 40 - 59 | 60 + |
| 2002 | 2 | 64 | 176 | 57 |
| 2003 | 2 | 52 | 140 | 45 |
| 2004 | 5 | 37 | 133 | 36 |
| 2005 | 1 | 39 | 154 | 64 |
| 2006 | 2 | 30 | 128 | 28 |
| 2007 | 1 | 10 | 80 | 33 |
| 2008 | 1 | 12 | 79 | 34 |
| 2009 | 1 | 9 | 68 | 41 |
| 2010 | 2 | 9 | 78 | 27 |
| 2011 | 0 | 11 | 67 | 30 |
| 2012 | 1 | 8 | 71 | 35 |
| 2013 | 0 | 10 | 35 | 33 |
| 2014 | 0 | 5 | 25 | 26 |
| 2015 | 0 | 4 | 18 | 23 |
| 2016 | 0 | 2 | 37 | 33 |
| 2017 | 0 | 4 | 27 | 32 |
| 2018 | 0 | 10 | 26 | 44 |
| 2019 | 0 | 3 | 28 | 28 |
| 2020 | 0 | 7 | 25 | 30 |
| 2021 | 1 | 6 | 20 | 37 |
| 2022 | 1 | 0 | 10 | 20 |
| 2023 | 0 | 12 | 11 | 35 |

**Table S8.** Number of estimated raw clonorchiasis cases in the dataset by major regions

|  | Major regions | | | | | | |
| --- | --- | --- | --- | --- | --- | --- | --- |
| Year | Seoul | Gyeonggi | Gangwon | Chungcheong | Jeolla | Gyeongsang | Jeju |
| 2002 | 27 | 26 | 1 | 22 | 45 | 174 | 4 |
| 2003 | 15 | 29 | 5 | 21 | 30 | 139 | 0 |
| 2004 | 19 | 23 | 2 | 21 | 33 | 111 | 2 |
| 2005 | 15 | 25 | 2 | 30 | 35 | 151 | 0 |
| 2006 | 9 | 15 | 4 | 23 | 23 | 114 | 0 |
| 2007 | 16 | 13 | 0 | 10 | 11 | 74 | 0 |
| 2008 | 12 | 16 | 1 | 8 | 14 | 73 | 2 |
| 2009 | 16 | 15 | 1 | 10 | 8 | 69 | 0 |
| 2010 | 9 | 19 | 2 | 7 | 12 | 67 | 0 |
| 2011 | 13 | 13 | 0 | 12 | 10 | 60 | 0 |
| 2012 | 9 | 10 | 0 | 7 | 11 | 77 | 1 |
| 2013 | 10 | 5 | 0 | 6 | 2 | 55 | 0 |
| 2014 | 7 | 7 | 1 | 3 | 8 | 28 | 2 |
| 2015 | 9 | 5 | 1 | 3 | 5 | 22 | 0 |
| 2016 | 5 | 9 | 0 | 4 | 6 | 47 | 1 |
| 2017 | 5 | 9 | 1 | 5 | 8 | 35 | 0 |
| 2018 | 8 | 7 | 3 | 6 | 17 | 38 | 1 |
| 2019 | 4 | 7 | 0 | 5 | 10 | 32 | 1 |
| 2020 | 9 | 10 | 0 | 7 | 7 | 28 | 1 |
| 2021 | 17 | 9 | 0 | 2 | 6 | 30 | 0 |
| 2022 | 7 | 3 | 0 | 3 | 3 | 14 | 1 |
| 2023 | 7 | 11 | 1 | 4 | 4 | 30 | 1 |

**Table S9.** Number of NHIS beneficiaries who received prescription at least once

| Year | National census | beneficiaries | Percentage |
| --- | --- | --- | --- |
| 2008 | 49,540,367 | 41,011,554 | 82.78 |
| 2009 | 49,773,145 | 42,350,733 | 85.09 |
| 2010 | 50,515,666 | 42,569,387 | 84.27 |
| 2011 | 50,734,284 | 43,025,242 | 84.81 |
| 2012 | 50,948,272 | 43,596,825 | 85.57 |
| 2013 | 51,141,463 | 43,774,781 | 85.60 |
| 2014 | 51,327,916 | 44,355,553 | 86.42 |
| 2015 | 51,529,338 | 44,507,236 | 86.37 |
| 2016 | 51,696,216 | 45,114,488 | 87.27 |
| 2017 | 51,778,544 | 45,235,327 | 87.36 |
| 2018 | 51,826,059 | 45,671,938 | 88.13 |
| 2019 | 51,849,861 | 45,730,440 | 88.20 |
| 2020 | 51,829,023 | 43,698,618 | 84.31 |
| 2021 | 51,638,809 | 44,023,295 | 85.25 |
| 2022 | 51,439,038 | 46,966,125 | 91.30 |
| 2023 | 51,325,329 | 46,916,852 | 91.41 |
